# eHealth Familias Unidas: Pilot Study of an Internet Adaptation of an Evidence-Based Family Intervention to Reduce Drug Use and Sexual Risk Behaviors Among Hispanic Adolescents

**DOI:** 10.3390/ijerph14030264

**Published:** 2017-03-04

**Authors:** Yannine Estrada, Lourdes Molleda, Ashley Murray, Kathryn Drumhiller, Maria Tapia, Krystal Sardinas, Alexa Rosen, Hilda Pantin, Tatiana Perrino, Madeline Sutton, Miguel Ángel Cano, Daphney Dorcius, Jessica Wendorf Muhamad, Guillermo Prado

**Affiliations:** 1 Department of Public Health Sciences, University of Miami, Miami, FL 33136, USA; l.molleda@med.miami.edu (L.M.); m.tapia1@med.miami.edu (M.T.); HPantin@med.miami.edu (H.P.); tperrino@med.miami.edu (T.P.); j.wendorf@med.miami.edu (J.W.M.); gprado@med.miami.edu (G.P.); 2 Division of HIV/AIDS Prevention, NCHHSTP, Centers for Disease Control and Prevention, Atlanta, GA 30333, USA; hfx8@cdc.gov (A.M.); kql6@cdc.gov (K.D.); zxa3@cdc.gov (M.S.); 3 Lifesource Biomedical, Herndon, VA 20170, USA; 4 Department of Epidemiology, Florida International University, Miami, FL 33199, USA; mcanojr@fiu.edu; 5 Herbert Wertheim College of Medicine, Florida International University, Miami, FL 33199, USA; ddorcius@med.miami.edu; 6 Department of Pediatrics, University of Miami, Miami, FL 33136, USA; k.sardinas@med.miami.edu; 7 4302 Snowberry Lane, Naples, FL 34119, USA; arosen@med.miami.edu

**Keywords:** Internet adaptation, Hispanic adolescent, substance use prevention, sexual risk behavior

## Abstract

This paper describes the Internet adaptation of an evidenced-based intervention for Hispanic families, eHealth Familias Unidas, and explores whether an Internet-based format is feasible and acceptable to Hispanic families. Core intervention components from the evidence-based intervention, Familias Unidas, were transposed into a video format and edited for content. Additionally, interactive exercises and a soap opera series were incorporated to reinforce intervention content and optimize participant engagement and retention. To understand the feasibility and acceptability of eHealth Familias Unidas, we conducted a pilot study and examined findings from: (1) session completion rates for both e-parent group sessions and family sessions (*n* = 23 families); and (2) qualitative data collected from Hispanic parents (*n* = 29) that received the eHealth intervention. Engagement and attendance in the intervention showed that 83% of families engaged in the intervention and that there was an overall session completion rate of 78%. Qualitative interviews were conducted mid and post intervention with a combined total of 29 participants. A general inductive approach was used to derive themes from the collected data. Overall, parents expressed positive feedback in regards to the intervention and stated that there were multiple lessons learned from participating in eHealth Familias Unidas. Findings indicate that an Internet-based family intervention is not only feasible and acceptable for Hispanic families, but also offers a viable option to ameliorate barriers to participation and implementation of preventive interventions.

## 1. Introduction

Health risk behaviors, such as substance use and sexual risk behaviors, among youth are critical public health issues because of their prevalence, early onset, and impact on multiple ecological systems, including family, school, and community [1]. Disparities in health risk behaviors continue to persist among racial and ethnic groups [2], including Hispanic adolescents. Hispanic 8th graders report the highest rates of use in nearly all classes of drugs compared to their non-Hispanic White and African American counterparts [3]. Furthermore, Hispanic high-school students are less likely to use condoms (55.6%) compared to their non-Hispanic White (56.8%) and Black peers (63.4%) [4]. Hispanic youth are also more likely to drink or use drugs before having sex (22.8%) compared to non-Hispanic White (19.3%) and black youth (21.8%) [4]. There is a clear need for preventive interventions to target the aforementioned disparities; not only because substance use and sexual risk behaviors are both related to the leading causes of death among adolescents [1], but also because Hispanics are a growing population in the United States. Hispanics are predicted to account for 29% of the U.S. population by 2060, a substantial increase from the current 17% [5,6]. Thus, the evaluation and dissemination of preventive interventions that have demonstrated efficacy and effectiveness in improving behavioral and mental health outcomes for Hispanics is a public health priority [7]. 

Currently, there is insufficient research on how to best implement and disseminate evidence-based preventive interventions for youth; therefore, many interventions never reach the targeted population. Further, the majority of evidence-based preventive interventions have not been evaluated with Hispanic populations. Research has suggested that family-based adolescent prevention strategies are a best practice for preventing substance use and sexual risk behaviors among Hispanic youth, yet family-based interventions have their own implementation and dissemination challenges [8,9,10]. Even at the efficacy stage, family-based interventions are plagued by barriers to participant engagement and attendance, two crucial elements to participants’ receipt of intended intervention dosage [11]. For example, attendance in family-focused interventions is usually low, with average attendance rates for universal parenting programs around 20% to 33% [12,13]. Other potential problems include fidelity to the intervention by trained facilitators in the community. Thus, the challenges affecting participant retention, community uptake, and program delivery have limited the forward progress of the field towards making evidence-based family preventive interventions available on a mass scale [14,15,16].

Internet delivered, or eHealth interventions, offer a viable option to ameliorate some of the barriers and challenges hindering the delivery of evidenced-based interventions [17]. eHealth interventions offer increased convenience for participants (e.g., they can log on to the intervention via mobile device or computer), and help maintain fidelity and cost effectiveness. In addition, eHealth interventions can expand access and availability to participants; research has shown that even low-income and minority populations have widespread access to the Internet [18]. Further, there is evidence that technology may play a critical role in substance use prevention through cost effective ways that facilitate fidelity and rapid, widespread adoption [19]. 

There is a growing body of literature showing the efficacy of eHealth interventions [20], including an increasing number of interventions that target parenting components as a way to impact adolescent problem behaviors such as drug use and sexual risk behaviors. For example, an eHealth intervention developed for mother–daughter dyads reduced risk factors, increased protective factors, and lowered alcohol use and intentions of use among African-American and Hispanic youth [21]. Studies have also found evidence of Internet-based intervention effects for depression [22] and substance use [23]. A review of parent and family focused eHealth interventions found significant reductions in behavior problems, depressive symptoms, parent-child communication and/or interaction, substance use, and relationship quality [24]. 

In terms of engagement, some research has shown predictors of engagement include level of family stress, acculturation, and effective parenting [17]. Little is known, however, regarding parent interest and willingness to view video-based content on parenting [25]. The limited body of research indicates that parents and youth have a preference for technologically-based behavioral interventions [25,26]. Ironically, the most commonly employed evidence-based approaches are in formats that are least preferred [25]. Although eHealth interventions have steadily increased in the last decade, few Internet-based programs have been developed that prevent drug use and sexual risk behaviors among Hispanic youth. Beyond the efficacy stage of clinical trial testing, few programs for culturally diverse adolescents have been evaluated [27]. Consequently, we have limited knowledge on how family-based eHealth programs are received among the Hispanic population. Therefore, the purpose of this manuscript is to describe the design of the Internet adaptation of the Familias Unidas intervention, called “eHealth Familias Unidas,” and the results from a feasibility and acceptability pilot study. 

Familias Unidas is a rigorously evaluated family-based preventive intervention designed to reduce drug use and sexual risk behaviors among Hispanic adolescents. Across four randomized trials [28,29,30,31,32,33], Familias Unidas has been shown to prevent and/or reduce substance use and/or sexual risk behaviors in Hispanic youth in addition to reducing conduct problems and improving family functioning [33]. The intervention is designed to empower parents to speak with their adolescent about the risks of substance use and sexual risk behaviors, while taking into account cultural variables that are unique to Hispanics. The face-to-face version of Familias Unidas consists of eight multi parent group sessions and four family sessions. The parent group sessions (consisting of 10 to 15 parents) are delivered by two facilitators, and the family sessions (consisting of a parent and their adolescent) are delivered by one intervention facilitator. During the parent group sessions, parents learn skills that are rehearsed through role-playing and are later applied in the family sessions, with guidance from one of the facilitators. Familias Unidas is based on ecodevelopmental theory, a contextual framework used to organize and address risk and protective factors for youth drug use and sexual risk behaviors from the macrosystem (i.e., the broad societal factors and philosophical ideals that define a particular culture, e.g., Hispanic cultural values and norms) to the microsystem (i.e., the contexts in which the adolescent participates directly, e.g., family) [34]. Further details about Familias Unidas can be found elsewhere [30].

Although nearly 20 years of rigorous investigation support Familias Unidas’ standing as a “program that works” (e.g., Substance Abuse and Mental Health Services Administration, Blueprints), Familias Unidas, like other family-based preventive interventions, faces challenges and barriers to engagement and participation including, transportation, scheduling, and time limitations (for both participants and facilitators) [35]. As a result, Familias Unidas intervention developers aimed to address barriers to parents’ participation, engagement, and retention, as well as the challenges of delivery, by adapting the program to be delivered through a more readily accessible medium: the Internet. 

This paper examines one overall research question: Is an Internet adaptation of the Familias Unidas intervention, eHealth Familias Unidas, feasible and acceptable to Hispanic families? By answering this question, this manuscript will provide insight into the use of the Internet for prevention among Hispanic populations. To understand the feasibility and acceptability of eHealth Familias Unidas, we examine findings from: (1) session completion rates for both e-parent group sessions and family sessions; and (2) focus groups conducted with Hispanic families that received the intervention.

## 2. Materials and Methods 

### 2.1. eHealth Familias Unidas Development and Components

The eHealth adaptation was developed utilizing the evidence-based Familias Unidas manual. The active components of the face-to-face group sessions were transposed into eight recorded e-parent group sessions. The intervention consists of eight online pre-recorded e-parent group sessions that are accessed via a website, and four parent-adolescent family sessions that are delivered by a trained facilitator. Sessions were designed such that parents were unable to fast forward or skip through sessions. However, we did not gate the intervention so that a forced sequential order was followed to prevent parents from watching an e-parent group session before a required family visit. There are two key differences between Familias Unidas and eHealth Familias Unidas: (1) Instead of a group of parents meeting with a facilitator, parents logged on to the eHealth Familias Unidas website to access pre-recorded e-parent group sessions. The e-parent group sessions consisted of video-recordings with three components: simulated parent group discussions, a culturally syntonic telenovela (soap opera) series, and interactive exercises. (2) Instead of a facilitator meeting with the family in person, the family and facilitator met online via web-based HIPPA compliant video-conferencing software (VSee; Table 1) [36].

Development of e-parent group sessions were theoretically grounded in entertainment-education (E-E), i.e., the process of creating and implementing a media message with the purpose of entertaining and educating to increase knowledge, create favorable attitudes, shift social norms, and modify behavior [37,38]. Entertainment-education can be viewed as a communication strategy to galvanize behavioral and social change [37]. E-E has demonstrated evidence in increasing knowledge about sexual health, including human papillomavirus [39] and condom use [40]. In regards to soap operas, in 1998, the Centers for Disease Control and Prevention established a program for education and research, acknowledging the importance of public health topics in soap operas [41]. As seen in the next section, tenets of E-E informed the simulated parent group discussions and the telenovela series. 

All intervention content was accessed through the eHealth Familias Unidas secure website; each participant was assigned a unique log-in name and password. The log-in procedure facilitated the close monitoring of participants, particularly as it related to session participation rates. Specifically, through the website, the research team was able to track who (i.e., which participant), when (i.e., day/time), how long (e.g., time of session attendance), and for what purpose (e.g., which session) the intervention website was accessed. No identifying participant information was placed on the website. The below sections provide more details regarding eHealth Familias Unidas program components. 

#### 2.1.1. Simulated Parent Group Discussions. 

Simulated parent group discussions consisted of enactments of the face-to-face group sessions. Parents from the local Miami community were recorded as they enacted each of the eight Familias Unidas group sessions. Participants in these enactments were Spanish-speaking, Hispanic parents with an adolescent between the ages of 12 and 16. Recordings were conducted in line with entertainment education theory which posits that to engage individuals with media, the receiver (i.e., person watching) needs to go through a process of identification and transportation [38]. Therefore, recordings depicted content that aimed to facilitate: (1) participant identification with the characters; and (2) the transportation of participants to the story line. Identification happens when the individual is connected to the characters (perhaps through perceived similarity) and transportation is when the individual is absorbed in the story. For these two elements to occur, the narrative must be persuasive and reduce resistance [38]. Hence, recordings were developed as close to reality as possible. Enactments were not scripted, parents were asked to proceed as if they were indeed in a Familias Unidas group and they were able to share their own personal struggles, provide support and give suggestions to each other as would take place in the intervention. Additionally, recordings were conducted in a manner to foster a sense of inclusiveness and interaction so that parents watching from home could feel they were part of the parent group as well. This was done by the facilitator regularly looking at the camera and asking parents at home questions such as: “For those of you watching at home, what are your thoughts on what your fellow group members are saying?” and “You watching at home, please think about what goals you have for your adolescent.” Given that each session in the original Familias Unidas intervention is approximately two hours long, recordings were edited, guided by the intervention manual, so that they succinctly delivered in approximately 15–20 min the important core intervention components that corresponded with each of the eight parent group sessions in the face-to-face Familias Unidas intervention.

#### 2.1.2. Telenovela Series

The second component consisted of a culturally syntonic telenovela series with eight episodes. Since the mid-1970s, research in the area of entertainment education [42] has explored the value of telenovelas and the impact telenovelas may have in addressing important social problems. Telenovelas represent an important, safe and neutral venue to discuss pressing social and health issues [43] that can present important public health topics. In addition, others have used telenovelas as a vehicle for public health campaigns and demonstrated their effectiveness [38,44]. Telenovelas are a persuasive behavioral change tool that can be used to increase engagement through reducing resistance to the message being delivered [45,46].

To create the telenovela series, we used scripts developed by an Emmy Award winning Hispanic scriptwriter, hired professional actors, and partnered with a university-based production team from the University of Miami School of Communication. To generate identification and transportation of participants to the storyline, the telenovela exemplified Hispanic cultural identity traits interwoven with real life events and fiction. Episodes were produced to be consistent with the intervention’s theoretical framework (e.g., placing parents in the role of authority and of educator for their youth). Episodes included scenes aimed at facilitating process conversations, improving parents’ understanding of the risks of drug use and sexual risk behaviors, improving parental understanding of the risks of drug use and sexual risk behaviors, and demonstrating ways youth can respond when faced with pressure to use drugs or engage in sexual risk behaviors. One episode was interweaved into each of the eight recorded parent group discussions to provide a scene directly related to the content covered in each of the eight parent group discussions, for example, adolescents’ risk for drugs use in the United States. For example, one sub-storyline follows a mother who is convinced that her son is drug-free and is shocked when he is arrested for involvement with drugs. Each telenovela lasted approximately ten minutes. The telenovela series was also used as an engagement strategy to keep parents interested in watching the parent group session component of the intervention. 

#### 2.1.3. Interactive Exercises

Interactive exercises were used to reinforce session content and to adapt the participatory learning strategy used in the face-to-face Familias Unidas intervention. As with the telenovela series, interactive exercises were interwoven within the parent group discussions. The type of interactive exercise varied from session to session and included true or false questions, multiple-choice questions, and point-and-click responses. Parents were provided with instant feedback regarding their responses and all on-screen text (e.g., instructions, response choices, answer summaries) was paired with audio to reduce literacy-related barriers. Intervention facilitators were able to review parents’ responses and use the gathered information to tailor discussion between parents and adolescents during the subsequent online family sessions according to family situations and needs. 

#### 2.1.4. Online Family Sessions

eHealth Familias Unidas facilitators were responsible for scheduling and delivering the four online family sessions (30–45 min) to each of their families. The content of each of the four family sessions was identical to that of face-to-face Familias Unidas, and provided parents the opportunity to practice the skills that they learned in the e-parent group sessions with their adolescent. Topics in the family sessions were couched according to the family’s needs and goals. Reminders for family sessions were delivered via phone or text message (according to parent preference) a few days before the scheduled family session. 

### 2.2. Pilot Study Procedures

eHealth Familias Unidas was pilot tested with parents utilizing an iterative process of participant feedback informing intervention modifications. A pilot study conducted in two phases tested the feasibility and acceptability of eHealth Familias Unidas. The purpose of Phase I (*n* = 6) was to test the intervention content, and not delivery of the intervention as would take place in a randomized trial. For this phase of the pilot study, six parents were brought to our research offices to watch the eHealth Familias Unidas intervention across a span of two weeks. Participants were asked to provide feedback regarding the intervention content and to provide suggestions about how the intervention could be improved. Phase II (*n* = 23) tested the intervention content, while also testing pilot procedures including recruitment strategies, flow of data collection procedures, and all technical aspects of the study in preparation for a full randomized controlled trial. For this phase of the pilot study, the full intervention was delivered to 23 families across a two-month period, including family sessions. Focus groups/interviews were conducted across both phases of the pilot study using the same questions. Recruitment and focus groups/interviews for both pilot studies were done through the Miami-Dade County Public School System (MDCPS) with IRB approval from the University of Miami Institutional Review Board and the MDCPS Research Review Committee (IRB number is 20110478). The inclusion criteria included: (a) adolescents of Hispanic origin; (b) adolescents in 8th grade at baseline; (c) adolescents living with an adult primary caregiver willing to participate; (d) families living within the catchment area of a MDCPS school at baseline; (e) families with access to the Internet (e.g., home, school, library); and (f) adolescents exhibiting a behavior problem as defined by MDCPS as Level I, II, and/or III behaviors. Level I included behaviors that disrupt the orderly operation of school functions such as cutting class, crude language; Level II are more serious, such as destroying property, confrontation with school staff; and Level III included offensive and harmful behaviors such as endangering safety, physically harming others. Families were not compensated for watching the intervention or completing the family sessions. 

### 2.3. Engagement and Participation

We define engagement as completion of at least one of the first three intervention sessions (i.e., completion of the first family session or viewing one of the first two e-parent group sessions [47]). Attendance was measured in terms of total attendance, completion of the family sessions, and viewing of the eight e-parent group sessions. The e-parent group sessions were tracked through programming in the eHealth Familias Unidas website while the family sessions were tracked through supervision sessions and submission of clinical documentation. e-Parent group sessions were marked as completed only if the participant finished all components of each session: fully watched the simulated parent group discussion and telenovela episode, and completed the interactive exercise(s). Engagement and attendance information was not collected for Phase I because its purpose was to gather information on intervention content, and not delivery of the intervention as in a randomized trial.

### 2.4. Facilitator Training and Supervision 

Facilitators for the eHealth Familias Unidas pilot consisted of Master’s level clinicians (e.g., social workers). All facilitators were fluent in Spanish and English. Facilitators consisted of three males and five females; all were of Hispanic descent. Facilitators were trained for two and a half days. The first two days consisted of clinical training involving delivery of the eHealth Familias Unidas family sessions. Facilitators received specialized training on how to engage and establish rapport with participants, didactic instruction about the intervention, clinical skills practice through role plays, and constructive review of recorded family sessions from previous Familias Unidas trials. Additionally, facilitators were required to watch the online e-parent group sessions, including completion of the interactive exercises. The last half day, facilitators were trained on how to access the online eHealth Familias Unidas intervention, how to trouble shoot difficulties with VSee, and how to track participant e-parent group session intervention completion through the eHealth Familias Unidas website. Throughout the course of intervention delivery, facilitators received a total of four group supervision sessions, each lasting approximately two hours. Supervision sessions were conducted online, with a few exceptions. Supervision sessions were used to review and obtain feedback on family sessions, troubleshoot any clinical concerns, and to review content of the intervention model. 

### 2.5. Focus Groups/Interviews

Focus group/interview participants consisted of 29 parents with an average age of 43 years (SD = 6.55). The vast majority of participants were female (*n* = 27). Given that the intervention is primarily delivered to parents, adolescents did not participate in the focus groups. 

#### Delivery

To obtain feedback from parents regarding the intervention, eight focus groups and six individual interviews were conducted with a total sample of 29 participants. Facilitators contacted assigned parents by telephone and offered them the opportunity to attend the focus groups/interviews. Parents were given a $60 gift card for their time. Four focus groups/interviews (*n* = 23 parents) took place halfway through the intervention after families watched group sessions one through four, and four focus groups/interviews (*n* = 16 parents) occurred at the end of the intervention when families completed watching all eight group sessions.

The focus groups/interviews were conducted at multiple middle schools that were relatively close to where the families lived and at the University of Miami offices. Each meeting lasted approximately an hour to an hour and a half. Examples of questions included: “What aspects/things in this session did you find you can use and that could be helpful or were not helpful?”, “What were some of your reactions to the telenovela?”, and “Are there things you would change that would make the intervention more appealing to you?” Additional questions sought to discover any technological issues parents may have experienced, comments on the interactive exercises, and whether weekly reminders from the facilitators to watch the sessions were reasonable.

Audio recordings of all qualitative data were transcribed verbatim and reviewed for accuracy. Audio recordings and transcriptions were stored in secure university servers. Transcriptions were uploaded into NVivo [48] and structural codes were applied to the data to label each focus group guide question and participants’ responses [49]. Two qualitative data analysts thoroughly read each transcript from start to finish and by structural code to create an emergent data-driven code list [50]. The analysts then met to discuss any discrepancies and to create a preliminary content codebook. To ensure coding consistency, analysts independently coded two transcripts and assessed inter-coder agreement using Kappa scores. Codes with a Kappa score less than 0.80 were reviewed and discussed until consensus was reached. Text segments were recoded and the codebook was finalized. Each analyst then coded six of the remaining twelve transcripts. Salient and co-occurring concepts were identified and organized into thematic categories.

## 3. Results

### 3.1. Engagement and Attendance

In Phase II, eighty-three percent (*n* = 19 out of 23) of participants engaged in the intervention (i.e., completed at least one of the following: first family session or one out of the first two e-parent group sessions). Seventeen percent of participants (*n* = 4 out of 23) engaged in zero sessions. Participants completed, on average, 9 out of 12 intervention sessions (SD = 4.64). Broken down into type of session, on average, participants completed three out of four family sessions (SD = 1.62) and six out of eight e-parent group sessions (SD = 3.10). Seventy percent of participants (*n* = 17) watched 100% of the e-parent group sessions and completed all four family sessions. 

### 3.2. Focus Group Themes

Two general themes emerged from the qualitative data: (1) parents expressed positive feedback in regards to the intervention; and (2) parents stated there were multiple lessons learned from engaging in eHealth Familias Unidas.

#### 3.2.1. Theme 1: Parents Provided Positive Feedback Regarding the e-Parent Group Sessions

Overall, parents in all 14 focus groups/interviews provided positive feedback about the intervention and stated that they would not change anything about the information presented. Parents praised the intervention and comments included “I loved it…” and “Thank you for this.” When asked how she felt about the information that was presented, one participating mother expressed a sense of empowerment in speaking with her son:

Everything is to educate them to contemporary life, open more eyes and educate parents so that we can also act better. I feel better now that I heard the videos, I feel safer talking to my son, with what tools I will begin, and I am very grateful to be invited and to learn more than what I had imagined I would learn. I did not give it too much importance, I am honest, at the beginning, but I said: "I’m going" and I liked very much because it has taught me a lot.

Another mother expressed positive views regarding the e-parent group session videos, “They are very nice, I liked everything that was presented. The project you made, I love it. It’s very acceptable, I liked it. Storylines in telenovelas and parents during group session were relatable.” What parents enjoyed the most was that the storylines featured in the telenovelas and the parents featured during the group discussions were relatable. The relatability of the storylines and parents was discussed in 12 out of the 14 focus groups/interviews. “To me all—well, from all of them I learned a lot and helped because there are things that I am not doing with my daughters, and this has made me learn, and reflect in some of the session. I saw myself reflected and—parents who were there were talking about, how you can accomplish it [discipline], that I feel will help me become better.”

#### 3.2.2. Theme 2: Lessons Learned as a Result of Being a Part of the Intervention (Viewing the e-Parent Group Sessions)

In addition to the positive feedback, the main content from all focus groups/interviews was related to the lessons that parents felt they learned from viewing the material. Four main lessons learned emerged: (1) effective parent–adolescent communication; (2) active parental attention and involvement in adolescent’s life; (3) importance of friends; and (4) importance of communication about sex. 

#### 3.2.2.1. Effective Parent-Adolescent Communication

Although effective communication skills were discussed during e-parent group session two, regardless of when parents took part in the focus groups (i.e., focus groups for sessions 1–4 or focus groups for sessions 5–8), the lesson of effective communication skills was discussed in all focus groups. Parents specifically noted that they learned strategies for listening and speaking to their children, and the importance of good communication. One mother noted the importance of respect and listening “I think listening to them, overall, listen first to what they are going to communicate to us, because sometimes we don’t hear or sometimes we listen, but we don’t hear, like she said [woman in video] on communication skills’. Yes. And if we are going to ask for something [of] them, or, to help as in housework, don’t scream, no… don’t go aggressively to ask them for a favor to help around the house, instead… most of all, speak with love, with respect to them, in order for them to respect us also.” Parents often discussed how they employed some of the communication skills they learned in the sessions:

I didn’t know the errors I was committing and thought: “I am one of that...” I am, well, strict, very strict, then my son says: “don’t love me too much because you are suffocating me.” And I love him so much that I want to be overprotective, and I know that at a certain extent it is bad. Our communication is the worst. We don’t communicate, but instead cry, fight, talk-back to one another and curse. And last night I liked that we had a moment with him, it was something insignificant but I liked it because I did not yell at him, and I started to say “wow.” I already saw a difference in 4 days.

Another mother shared a change in how she communicates with her son, “So, at least it’s helped me a lot, because now I do listen to my son, although it’s still a little difficult for me, but I try, I don’t stop there, instead I do try…”

#### 3.2.2.2. Parental Attention and Involvement in Adolescent’s Life

By viewing the e-parent group sessions, specifically e-parent group session one, parents learned the importance of paying attention to their children and being actively involved in their lives. Comments included, “we have to give them support” and “we have to get involved.” The importance of attention and involvement was discussed in 13 out of the 14 focus groups/interviews. One mother spoke of supporting youth in “their goals, for instance, some children are mature, but there are others who sometimes need the support of the parents so that they can choose a career or a job. Sometimes parents, such as the one soap opera, that the mother did not support their children. And sometimes, that causes that child to lose focus.” Another mother reflected on seeing herself in the video when she does not listen to her son: “Sometimes, not all, but in many things from the video, the soap opera, there are things that are reflected to us. As it [is], often, we do not pay attention, and when they come to us to say something like, ‘I want to talk,’ and one says, ‘no, I’m busy, I’ll speak with you later.’” A father participant discussed how sometimes busy lives can interfere with adolescent involvement and get in the way:

I think that, really, as parents sometimes, since we’re running around daily with the system here of this country, one doesn’t take time to, really, to get involved with them, on what’s going on with them, what’s happening in their daily lives. So then, well, that video like illustrates that, you know? That one has to be interested and talking to them and pay attention, and get more involved in their personal lives. What is it that they feel, what they like, what they don’t like.

Similar to employing effective communication skills, parents also began to “pay more attention” to their children after viewing the e-parent group sessions. “Well, lately I am putting more attention, I pay more attention when my daughter speaks to me, because I stop what I am doing and look at her, I hear her.”

#### 3.2.2.3. Importance of Friends

In 11 focus groups/interviews, parents discussed how important it is that they are aware of their children’s friends. By viewing the e-parent group sessions, parents learned both the positive and negative effect friends can have, as depicted in e-parent group session six. “How to resist the pressure from friends, explain to them well those things. There are good friends that are of good influence and others who are not. How to help them with those friends, and share with them the experience of each one which is very important nowadays.” Parents also discussed the importance of getting to know their children’s friends, “What’s important is knowing the type of friends our children have. Because, sometimes, that—there are some who give very negative influences to the kids. And we, I mean, we’re at work, not paying attention to them, not knowing what they do, who they’re with, and that has a lot to do with it.”

#### 3.2.2.4. Importance of Communication about Sex

In eight focus groups/interviews, parents stated that by viewing the e-parent group sessions, i.e., session seven, they learned about the importance of discussing sex with their children and how to do so. Parents specifically learned how to discuss condoms and HIV. When asked her reactions to the session, one mother explained, “we should try to talk to our children and entering the issue [sex] carefully to make them feel comfortable, at the right time, and being alone if it is possible to make them feel comfortable that someone is watching them and they have no confidence to relax and communicate their concerns… And so that’s the danger when they challenge us, and we have not spoken with them. And you have to be preventive during all those dangers that surround us in general.” Parents focused on the importance of preparing youth on how to properly use a condom, “And also I note something that, when we talked about the prevention of AIDS, they should also be prepared to know about the use of condoms. That part was very good. That if you have the bubble, if you have expiration date... how to put it [condom] on, how to do so, how to pull it out, tie it and discard. Prevent any STD, not only AIDS, there are many more than that.” Parents also reported the importance of having family rules as a means of protecting youth, “Talk to them, and teach them how dangerous the HIV virus, advise them, and how to say... have rules to tell them that when they take friends to the house, not to close the door… and for me there has to be rules for this reason.”

### 3.3. Intervention Modification and Challenges 

Modifications were made to the e-parent group sessions based on participant feedback from the focus groups. Parents stated that the audio was off; therefore, it was equalized to reduce volume level fluctuations, an interactive exercise was altered to remove errors in the text, and the opening “jingle” for the e-parent group sessions was changed to a new tune because parents stated it was too jovial for the serious nature of topics being covered. Additionally, a handful of aesthetic changes were made, per participant request. For example, the quality of the e-parent group session graphics (e.g., bar graphs, icons, and text) were increased through improved resolution. To help families stay on track with watching the e-parent group sessions, a reminder text message was sent to participants once a week. Additionally, a three-minute tutorial video was developed to help participants with any log in difficulties. Modifications were also made to the website to centralize a location where facilitators could go to request information about their families and to receive technical support. Intervention-related forms (e.g., recruitment sheets, call logs, etc.) were uploaded to the website for easy and quick access for facilitators. To help facilitators keep track of all these features, “pocket guides” were created with simple instructions on how to access the website and where to access forms. 

Challenges in evaluating the feasibility of this study revolved around technical barriers. For example, some parents had difficulty downloading the VSee software onto their systems, requiring assistance from the facilitator or a staff member with technical expertise. Additionally, some facilitators and families had challenges regarding slow Internet speed causing audio delays during the family sessions. This required training on how to manage these situations from a technical and engagement point of view to minimize frustration. In addition, families that needed a webcam were provided with one to be able to interact with the facilitator.

## 4. Discussion

The current study showed that: (1) an evidence-based family intervention can be adapted for use in an eHealth format; and (2) it is feasible to recruit, engage and retain Hispanic families into an eHealth intervention, and deliver it electronically. Findings from session completion rates and focus groups/interviews conducted with Hispanic families that received the intervention in the pilot study indicate that eHealth Familias Unidas is feasible and acceptable, therefore adding to the limited body of research on computer mediated interventions with minority populations. Poor participation rates are a perennial problem for preventive interventions [11,16,51]. eHealth interventions can capitalize on the widespread use of the Internet to reach underserved groups and reduce intervention delivery costs associated with disseminating evidence-based interventions [20]. eHealth Familias Unidas holds promise as a viable option to help ameliorate the traditional challenges of family participation in face-to-face preventive interventions by providing engaging, culturally appropriate online content, allowing parents to access sessions at their convenience, and minimizing costs for researchers and participants alike.

In contrast to some Internet-based interventions [52,53], we experienced strong engagement and retention with 83% of participants engaging in the intervention and a mean of 78% of total sessions completed. Participant positive perceptions of the intervention, as reported in the focus groups/interviews, mirrored the engagement and participation rates in Phase II of the pilot study. The high attendance is a testament to the engaging nature of the online sessions, which may be attributed to the cultural specificity of our content for Hispanic families. For example, qualitative data indicated that parents in the video group discussions were relatable and participants identified with the storylines in the telenovela series. The high engagement and participation rate also provides information regarding Hispanic parent interest in and willingness to view video messages about parenting, which was found to be a preferred format in another media-based parenting intervention [25]. Furthermore, as seen in the focus groups/interviews, participants found value in the lessons learned via the intervention sessions which may also positively impact parent engagement with eHealth Familias Unidas. Specifically, parents highlighted learning about the importance of effective communication, being attentive and involved with their youth, and communicating about sex. 

While the corpus of our findings highlight the feasibility and acceptability of eHealth interventions with Hispanic populations, it is important to note that eHealth interventions have their own challenges [54]. For example, participants may not log on to the website to view the intervention sessions, or may only watch part of the sessions, which inhibits participants from receiving the intended intervention dosage. In addition, some researchers have noted that online interventions do not give the participant an opportunity to interact or practice their skills, which may limit the participant from applying the information learned to their daily lives. We attempted to mitigate the aforementioned challenges through interactive and participatory sessions; the interactive exercise component challenged the parent to think through how the content in the parent group session and telenovela may be applied to their family. With guidance from a trained facilitator, the family sessions were an opportunity for the parent to enact the skills learned in the group sessions with their adolescent. Indeed, research with eHealth interventions has indicated better participant adherence for eHealth interventions with a human component that offers “supportive accountability” [17,53,55]. Supportive accountability includes the presence of a coach that creates accountability to adherence goals, monitors progress, establishes a bond and is seen as trustworthy [17,53,55]. Over the course of the intervention, the facilitator formed a trusting relationship with the families, which helped to hold the families accountable, and engaged them to continue participating in the family sessions and logging on to view the e-parent group sessions. 

This study has some limitations that should be acknowledged. First, given that this a feasibility and acceptability study, it is not possible to make any conclusions regarding the efficacy of the eHealth Familias Unidas intervention. Currently, there is an ongoing randomized trial with eHealth Familias Unidas that will determine whether the intervention has an impact on family functioning as well as youth health risk behaviors. Second, the sample for this study came from a single geographic area, Miami-Dade County, which may not be representative of Hispanics in the United States. Finally, there is an inherent self-selection bias with participants who decide to participate in the focus groups; there may be differences between families that decide to participate and those that do not. Further, a selection bias also exists with our eligibility criteria, which required families to have Internet access, therefore, potentially excluding families from low socioeconomic status. However, this eligibility criterion was necessary to ascertain that families would be able to access the intervention.

## 5. Conclusions

In conclusion, given the public health need to reach and decrease behavioral health disparities affecting the Hispanic population, eHealth Familias Unidas offers an accessible, potentially cost-effective, and culturally acceptable option for families aiming to prevent problem behaviors in their youth. Given that the main goal in this study was to evaluate feasibility and acceptability, not to test efficacy, the next logical step is to test the efficacy of eHealth Familias Unidas before disseminating on a larger scale. Two studies are currently testing the efficacy of eHealth Familias Unidas in the school system and in four primary care clinics in South Florida. These efficacy trials will explore cost-effectiveness, provide more information on the acceptability of eHealth Familias Unidas, and examine intervention effects, including family functioning and youth risk behaviors (e.g., substance use and sexual risk behaviors). Participant feedback will also be gathered from the two trials to continue to inform the improvement and evolution of eHealth Familias Unidas.

## Figures and Tables

**Table 1:** eHealth Familias Unidas session overview.

Session #	Session Title	Session Content
1	Family Session #1 Engagement and Orientation	Engagement with parents and youth. Completion of family needs assessment and problem-solve family’s perceived barriers to participation.
2	e-Parent Group Session #1 Parental Investment in Adolescent Worlds	Provides an introduction to eHealth Familias Unidas and a review of adolescent risk factors. Parents engage in an interactive exercise to set goals in each of the adolescent’s worlds (i.e., family, school, and peer).
3	e-Parent Group Session #2 Enhancing Communication Skills	Focuses on the characteristics of effective family communication and parents engage in an interactive exercise to reinforce key communication skills.
4	Family Session #2 Family Communication	Parents use newly learned communication skills and practice with adolescents by discussing a relevant issue in the youth’s life.
5	e-Parent Group Session #3 Family Support and Behavior Management	Highlights the significance of parental support, behavior management, and effective discipline. Parents complete an interactive exercise that reinforces behavior management strategies.
6	e-Parent Group Session #4 Parental Monitoring of Peer World	Highlights the role of prosocial and antisocial peers and the protective effects of parental monitoring. Parents complete an interactive exercise that reinforces parental monitoring strategies.
7	e-Parent Group Session #5 Adolescent Drug Use	Focuses on the prevalence and consequences of adolescent drug use. Parents complete an interactive exercise on strategies to prevent adolescent drug use.
8	Family Session #3 Parental Monitoring of Peer World and Adolescent Drug Use	Covers family conversations about adolescent’s peers. Provide ways to troubleshoot interactions between parents and the youth’s peer world. Parents teach youth the skills necessary to effectively manage peer pressure to engage in drug use.
9	e-Parent Group Session #6 Parental Investment in Adolescent’s School	Addresses the role of school in the adolescent’s life and how parental connections to school can serve as a protective mechanism. Parents engage in an interactive exercise that emphasizes parental involvement in the adolescent’s school world.
10	e-Parent Group Session #7 Adolescent Sexual Risk Behaviors	Discusses parental attitudes and beliefs regarding adolescent sexual risk behaviors, the effects of adolescent sexual risk behaviors on STIs, and what parents can do to influence adolescent behavior. Parents complete two interactive exercises to reinforce knowledge of risky sex and safe sex practices.
11	Family Session #4 Adolescent Sexual Risk Behaviors	Parents communicate the dangers and consequences of sexual risk behaviors. Parents guide their adolescent in developing safety skills.
12	e-Parent Group Session #8 Prevention Has To Be Achieved All Over Again Everyday	Review the content of eHealth Familias Unidas. Highlight parents’ role as lifetime educators of their adolescent and the importance of daily implementation of skills to improve family functioning in order to prevent drug use and sexual risk behaviors.
